# AI‐Enhanced Diagnosis of Challenging Lesions in Breast MRI: A Methodology and Application Primer

**DOI:** 10.1002/jmri.27332

**Published:** 2020-08-30

**Authors:** Anke Meyer‐Base, Lia Morra, Amirhessam Tahmassebi, Marc Lobbes, Uwe Meyer‐Base, Katja Pinker

**Affiliations:** ^1^ Department of Scientific Computing Florida State University Tallahassee Florida USA; ^2^ Department of Radiology, Maastricht Medical Center University of Maastricht Maastricht Netherlands; ^3^ Department of Control and Computer Engineering Politecnico di Torino Torino Italy; ^4^ GROW School for Oncology and Developmental Biology Maastricht Netherlands; ^5^ Department of Electrical and Computer Engineering Florida A&M University and Florida State University Tallahassee Florida USA; ^6^ Department of Radiology, Breast Imaging Service Memorial Sloan‐Kettering Cancer Center New York New York USA; ^7^ Department of Biomedical Imaging and Image‐Guided Therapy, Division of Molecular and Gender Imaging Medical University of Vienna Vienna Austria; ^8^ Zuyderland Medical Center, dep of Medical Imaging Sittard‐Geleen Netherlands

**Keywords:** computer‐aided diagnosis systems, machine learning, kinetic features, morphologic features, magnetic resonance imaging, breast cancer

## Abstract

Computer‐aided diagnosis (CAD) systems have become an important tool in the assessment of breast tumors with magnetic resonance imaging (MRI). CAD systems can be used for the detection and diagnosis of breast tumors as a “second opinion” review complementing the radiologist's review. CAD systems have many common parts, such as image preprocessing, tumor feature extraction, and data classification that are mostly based on machine‐learning (ML) techniques. In this review article, we describe applications of ML‐based CAD systems in MRI covering the detection of diagnostically challenging lesions of the breast such as nonmass enhancing (NME) lesions, and furthermore discuss how multiparametric MRI and radiomics can be applied to the study of NME, including prediction of response to neoadjuvant chemotherapy (NAC). Since ML has been widely used in the medical imaging community, we provide an overview about the state‐of‐the‐art and novel techniques applied as classifiers to CAD systems. The differences in the CAD systems in MRI of the breast for several standard and novel applications for NME are explained in detail to provide important examples, illustrating: 1) CAD for detection and diagnosis, 2) CAD in multiparametric imaging, 3) CAD in NAC, and 4) breast cancer radiomics. We aim to provide a comparison between these CAD applications and to illustrate a global view on intelligent CAD systems based on machine and deep learning in MRI of the breast.

**Level of Evidence:**

2

**Technical Efficacy Stage:**

2

BREAST CANCER is the most common cancer among women but has an encouraging cure rate if diagnosed at an early stage. Thus, early detection of breast cancer continues to be key for effective treatment. Magnetic resonance imaging (MRI) is an established essential tool in breast imaging for high‐risk screening, assessment, diagnosis, staging, and follow‐up of breast cancer.^1^, ^2^ It has a proven value in important areas such as evaluating local extent of disease, multicentricity, response to neoadjuvant chemotherapy, and in the assessment of the integrity of implants.^1^, ^3^ Currently dynamic contrast‐enhanced MRI (DCE‐MRI) is the most sensitive imaging technique for breast cancer diagnosis with a high specificity, is independent of breast density, and detects noninvasive breast cancer. The limitations in specificity can be overcome by employing additional functional MRI techniques such as diffusion‐weighted imaging (DWI) and proton MR spectroscopy. These techniques have demonstrated an improved diagnostic accuracy as well as response assessment^4^; their combined application is called multiparametric MRI and can be utilized for the detection and characterization of breast tumors.^5^, ^6^ However, the acquisition of multilayered multidimensional data poses new challenges to radiologists; and thus new tools for reliable, reproducible, and quantitative assessments are warranted for improved diagnosis, tumor characterization, and treatment monitoring.

Inspired by computer‐aided diagnosis (CAD) systems to support diagnostic and screening activities in conventional X‐ray mammography, research initiatives nowadays focus on similar techniques to aid or even automatize the diagnosis in MRI of the breast. These efforts started as early as 2002 with the application of the multilayer perceptron (MLP) as a classifier of tumor‐extracted features describing dynamic, morphological, or combined characteristics^7^, ^8^, ^9^ for breast MR segmentation and lesion detection,^10^ while achieving results comparable to that of an expert radiologist. Other machine‐learning (ML) techniques used in the early days were the fuzzy c‐means clustering‐based technique for automatically identifying characteristic kinetics from breast lesions^11^ and the mean shift clustering for determining accurate regions of interest (ROIs) in breast MRI lesions.^12^ All these techniques for CAD systems of mass lesions outperformed an experienced radiologist and demonstrated that ML techniques can support the radiologist in the diagnosis of breast lesions.

However, the delineation, detection, and diagnosis of nonmass enhancing (NME) lesions is clinically very challenging since the standard morphological and kinetic features that are relevant for masses fail to achieve equally good results for NME lesions.^13^ Thus, CAD systems strongly relying on morphological and kinetic parameters are proven to be insufficient to obtain a satisfactory performance for NME lesions. Designing robust and reliable CAD systems for these NME lesions represents a challenge for the medical imaging specialist. Few studies in the literature explore CAD systems based on ML techniques are reported for this type of lesion.

To provide useful insights for ML techniques in connection with important CAD systems of NME lesions in MRI of the breast, this review article consists of seven sections. Section *Application of ML and Dynamic Contrast Enhancement (DCE) Kinetics of Breast Tumors* describes the basic kinetics aspects in breast MRI, while Section *Application of ML and Morphology of Breast Tumors* explains the equally important morphological criteria used in detection and diagnosis. Section *Machine Learning Techniques* provides an overview about the most important standard and novel ML techniques and their processing steps. The next sections present applications of ML to relevant topics in MRI of the breast and include all necessary preprocessing steps to achieve a diagnostic solution. Section *CAD in MRI of the breast* presents intelligent diagnostic solutions based on tumor‐extracted features and enhancement curves. Section *Future Trends: CAD Systems for Novel Applications* covers future trends including novel applications of ML in MRI of the breast.

## Application of ML and Dynamic Contrast Enhancement (DCE) Kinetics of Breast Tumors

**FIGURE 1:**
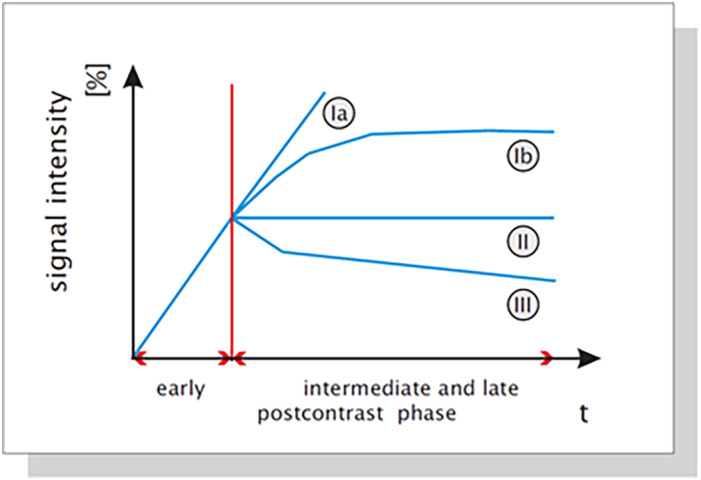
Schematic drawing of the time–signal intensity (SI) curve types. ^KMK+ 99^ Type I corresponds to a straight (Ia) or curved (Ib) line; enhancement continues over the entire dynamic study. Type II is a plateau curve with a sharp bend after the initial upstroke. Type III is a washout time course. In breast cancer, plateau or washout–time courses (type II or III) prevail. Steadily progressive signal intensity time courses (type I) are exhibited by benign enhancing lesions.

As mentioned in the previous section, morphologic, kinetic, or combined features represent important lesion characteristics for a computer‐assisted interpretation. For example, time–signal series, as measured during a DCE‐MRI examination for each image voxel, represents an important component in designing CAD systems for MRI of the breast. Early studies have demonstrated that the shape of the time–signal intensity curve provides an important biomarker for discriminating between benign and malignant enhancing lesions in DCE‐MRI and it is a key step of reporting MRI of the breast.^14^ It has been shown that the enhancement kinetics, as represented by the time–signal intensity curves, visualized in Fig. 1, differ significantly for benign and malignant enhancing tumors and thus are representative of differential diagnosis: plateau or washout‐time courses (type II or III) are mostly found in cancerous tissue. Steadily progressive signal intensity time courses (type I) are typical of benign enhancing lesions. Typical features representative of kinetics are maximum enhancement, time to peak, uptake rate, washout rate, enhancement at first postcontrast timepoint and signal enhancement ratio.

Recently, new *k*‐space acquisition strategies have been introduced for dynamic breast MRI such as time‐resolved angiography with stochastic trajectories (TWIST) and differential subsampling with Cartesian ordering (DISCO).^15^, ^16^ These ultrafast sequences can be used to capture the inflow of contrast in breast lesions, heavily undersampling the outer part of the *k*‐space in order to increase the spatial resolution for an improved diagnostic quality. Thus, it can be employed in clinical settings to acquire breast DCE‐MRI data with both high spatial resolution for accurate tumor morphology assessment and high temporal resolution for accurate representation of the contrast agent kinetics.^2^, ^17^ The potential of these new breast MRI techniques for screening and automated characterization of breast lesions has not yet been explored. A single study^2^ has shown that the ultrafast protocol yielded a high diagnostic accuracy compared with the standard protocol when the maximum slope of the relative enhancement vs. time curve (MS) was used as a kinetic information vs. the Breast Imaging‐Reporting and Data System (BI‐RADS) curve types. A more precise evaluation can be achieved based on advanced computer tools that could additionally incorporate the morphologic information and assist the radiologist in image interpretation and patient workup.

There is clinical evidence that novel enhancement curve parameters combined with morphological features are improving the diagnostic accuracy for the ultrafast protocol.^18^

Although signal characteristics represent an important biomarker for a radiologist to distinguish between different tissue states, their assessment is quite a time‐consuming task. This becomes challenging when the heterogeneity of lesion tissue is considered, which causes the spatial variation of signal characteristics. In addition, this variation reflects specific tissue properties that should be considered when assessing the state of lesions. Kinetic parameters extracted either from qualitative BI‐RADS or quantitative empirical mathematical models measures of kinetics have proven to be insufficient when it comes to the differential diagnosis of NME lesions.^19^

## Application of ML and Morphology of Breast Tumors

Morphological parameters describing either the shape or structure of the ROI are obtained from manual or semiautomatic detection. They are either qualitatively or quantitatively extracted from lesions and represent valuable diagnostic biomarkers.^20^ The most important morphological features are area, compactness, perimeter, smoothness, radial length, roughness, sphericity, volume, spiculation, curvature, and edge. While qualitative morphological features have a high interobserver variability,^21^ quantitative ones provide a more standardized and objective diagnosis. Other nonkinetic features besides the morphological are histogram features, spiculation, textural, geometric, and binary object features. Since the NME lesions exhibit ambiguous characteristics when limited to only dynamical or morphological parameters alone, a fusion of different dynamic and morphologic characteristics proved beneficial in terms of diagnostic sensitivities and specificities.^22^, ^23^

**FIGURE 2:**
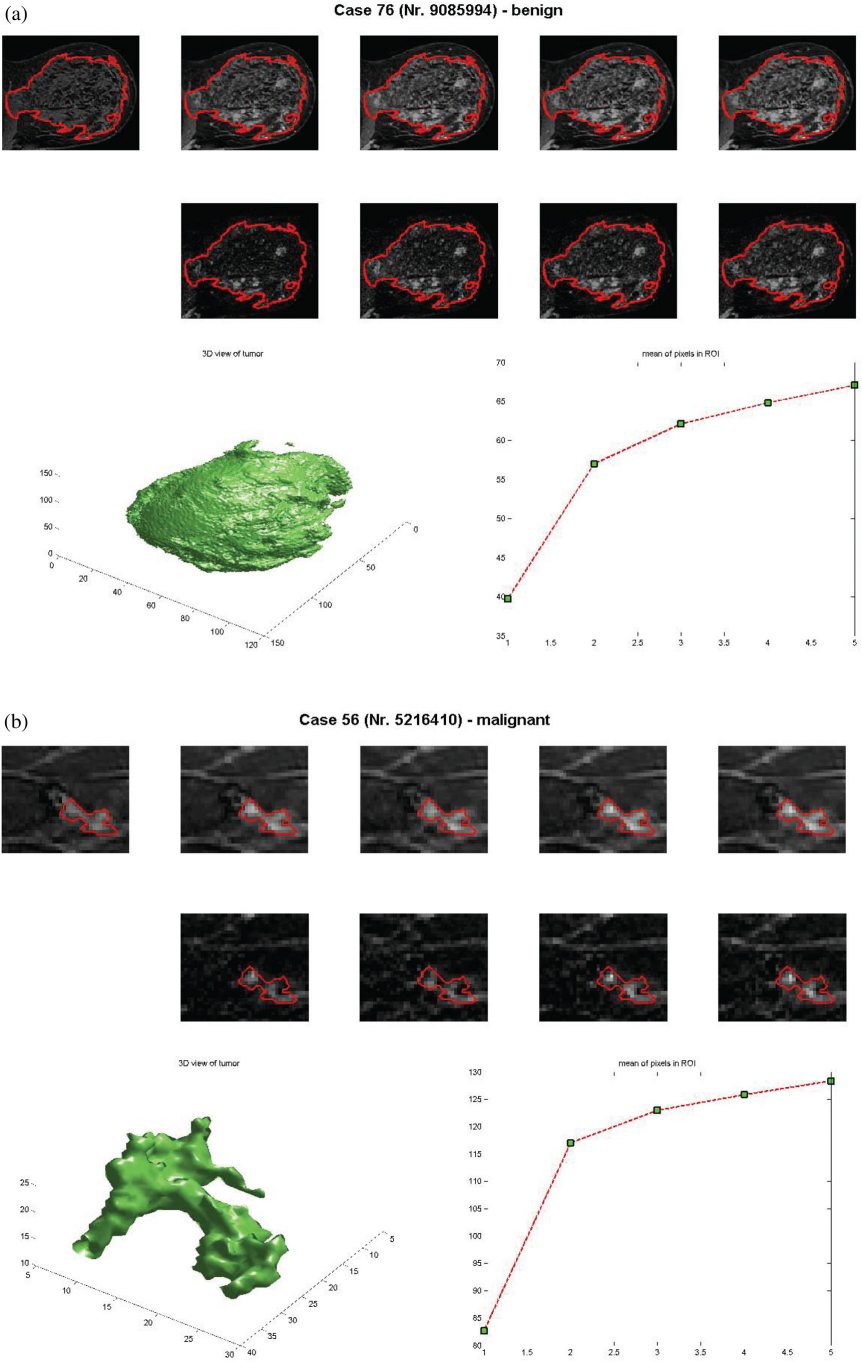
Morphological and dynamic representations of segmented benign (diffusely enhancing glandular tissue) and malignant (invasive ductal carcinoma) nonmass‐like‐enhancing lesions. The time‐scans in the first row are without motion compensation, while those in the second row are motion‐corrected. The left image in the last row shows the segmented tumor, while the right one shows the SI curve.

Based on morphology and type of enhancement, lesions are assigned according to risk assessment and a quality assurance tool, the BI‐RADS lexicon, to mass enhancement, nonmass, and focus.^13^, ^24^, ^25^ Masses are 3D tumors that have either a round, oval, lobular, or irregular shape; nonmasses have poorly defined boundaries and considerable overlap in kinetic characteristics between malignant and benign lesions; and foci represent small spots of enhancement that cannot be characterized as a mass. The diagnosis of mass enhancement lesions is straightforward and employs typical characteristic parameters such as spiculation (morphology), rim enhancement (texture), and washout kinetics. However, the diagnosis of foci and nonmass‐like enhancing lesions pose a challenge to both clinical reading and CAD systems. Therefore, standard parameters cannot be applied, and novel image and signal processing techniques need to be developed and integrated into the CAD system. While for mass‐enhancing lesions several BI‐RADs descriptors are used for the differential diagnosis, the existing BI‐RADS descriptors for NME lesions have proven to be insufficient for the automated differential diagnosis. Nonmass‐enhancing lesions represent a diagnostic challenge in MR, as exemplified in Fig. 2.

The correct detection of these lesions, in combination with many clinical applications ranging from diagnosis to therapeutic solutions, demand sophisticated image processing paradigms in connection with feature extraction. The response to these challenging processing tasks has guided the development of novel ML techniques, which will be described further below.

## Machine‐Learning Techniques

Several ML techniques are incorporated as a classifier in CAD pipelines for breast cancer detection, prediction of neoadjuvant chemotherapy outcome, and diagnosis. A brief description of the most important techniques is given in this section. We start with “classical” ML approaches such as support vector machine and random forest and conclude with a brief discussion of deep learning.

### Artificial Neural Networks Classifiers

Artificial neural network (ANN) classifiers are an attempt to emulate the processing capabilities of biological neural systems. The architecture of the MLP is completely defined by an input layer, one or more hidden layers, and an output layer. Each layer consists of at least one neuron. The input vector is processed by the MLP in a forward direction, passing through each single layer. A neuron in a hidden layer is connected to every neuron in the layers above and below it. MLPs have been applied successfully to sophisticated classification problems. The training of the network is accomplished based on a supervised learning technique that requires given input–output data pairs. The training technique, known as the error backpropagation algorithm, is bidirectional, consisting of a forward and backward direction. During the forward direction a training vector is presented to the network and classified. The backward direction consists of a recursive updating of the weights in all layers based on the computed errors.

### Random Forests

The random forest represents a powerful statistical learning technique and is an ensemble method^26^ composed of many smaller models. The classification and prediction are achieved by combining the outputs of these smaller models that are usually classification and regression trees (CART). CART operates based on a repeated partitioning of the input data in order to estimate the conditional distribution of a response (output class) for a given set of feature variables. The algorithm implements a binary decision tree where every single feature of the input is considered a candidate for the split. Binary decision trees are nonlinear multistage classifiers. This classification system operates by rejecting sequentially classes until the correct class is found. In other words, the correct class corresponding to a feature vector is determined by searching a tree‐based decision system. The feature space is divided into regions corresponding to the different classes. The goal of CART is that the emerging subgroups after the split are homogeneous. The trees are combined to a forest based on bagging. The variance of the predictions of a model is decreased by fitting several models and then taking the average over their predictions to achieve in the end a regularized prediction. To avoid overfitting, each model is fitted only to a sample of the same size as the original input data but selected with replacement. This sample technique is known as the bootstrap sample.

### Support Vector Machines

The support vector machine (SVM) represents a feedforward single layer classifier that can be employed either for linear or nonlinear separable datasets.^27^, ^28^, ^29^ It became for many classification problems in biomedical imaging the first‐choice classifier.

The basic idea of the SVM algorithm is to design a hyperplane:

(1) $$fw,b\left(x\right)=w\cdot x+w0$$

characterized by its direction vector w ∈ R^*n*^ and its exact position in space or bias *w*0. The hyperplane separates the labeled input or training data into two classes by leaving the maximum‐margin from both classes. A given set of *N* labeled training examples {(x, *y*)*i*}, *i* = 1,…, *N*, x*i* ∈ *R* ^*n*^ assigned to two different classes *yi* ∈ {−1, 1}, is separated by a maximum‐margin hyperplane such that the distance between the hyperplane and the closest examples (the margin *γ*) is maximized. This hyperplane is fully specified by a subset of those training examples that lie closest to the decision surface and pose a challenge for a correct classification. The training samples that lie closest to the hyperplane represent the support vectors. To employ the SVM for nonlinearly classifiable data, we need to employ the so‐called “kernel trick.” This symmetric and nonlinear kernel function evaluates the inner product between two examples after their transformation by a nonlinear function by maintaining the original architecture of the linear SVM.

### KNN Classifier

K‐nearest neighbors (KNN) is a supervised classifier. This algorithm stores all available patterns and classifies new patterns based on a similarity measure (eg, distance functions). This procedure can be very easily elucidated based on a two‐class classification task: an unknown pattern x should be assigned to one of the two classes *C*1 or *C*2. The decision is made by determining its Euclidean distance *d* from all the trainings vectors belonging to various classes. We define two hyperspheres with the radius *r*1 and *r*2, respectively centered at x. Let *V*1 and *V*2 be the two hypersphere volumes corresponding to the two classes *C*1 and *C*2.

The *k‐*nearest neighbor classification rule can be easily formulated in the case of two classes *C*1 and respectively *C*2 as:

(2) $$\text{Assign}\;x\;to\;\text{class}\;C_{1}\;\left(C_{2}\right)\;\text{if}\;\frac{V_{2}}{V_{1}}>\left(<\right)\frac{N_{1}P\left(C_{2}\right)}{N_{2}P\left(C_{1}\right)}$$

### Bayesian Classifier

Bayes decision theory represents a fundamental statistical approach in pattern classification assuming mutually exclusive and exhaustive classifications with known prior probabilities. Simplified formulated, the probability that a pattern belongs to a given class is determined.

A simple example represents the two‐class case with *C*1, *C*2. The a priori probabilities *P* (*C*1) and *P* (*C*2) are assumed to be known a priori since they can be easily determined from the available training dataset. Given are the pdfs *p*(x*i*|*Ci*), *i* = 1, 2. These pdfs *p*(x*i*|*Ci*) are known as likelihood functions of *Ci* with respect to x.

The Bayes classification rule can be easily stated for the two–class case *ω*1, *ω*2 as:

(3) $$\text{If}\;P\;\left(C1|x\right)>P\;\left(C2|x\right),\;x\;\text{is assigned to}\;C1$$

$$\text{If}\;P\;\left(C1|x\right)<P\;\left(C2|x\right),\;x\;\text{is assigned to}\;C2$$

Based on the above classification algorithm, a feature vector can be either assigned to one class or the other. This is equivalent to determining the maximum of the conditional pdfs evaluated at x.

### Bayes Classification Based on LDA and QDA

As stated before, the Bayes classification^30^ is based on determining the prior probabilities *πi* for each class *Ci*. This value describes the prior estimates about how probable a class is.

This classification method assigns each new training sample to the class with the highest posterior probability.

Thus, the classification rule becomes:

(4) $$Cj={\left(xi-\mathrm{\mu j}\right)}^{T}{\left(\sum j\right)}^{-1}\left(xi-\mathrm{\mu j}\right)+\log\;|\sum j|-2\;\log\mathrm{\pi j}$$

where *μj* represent the means of the classes and ∑*j* is the corresponding covariance matrix. The assignment to a certain class *j* for a certain input pattern is made based on the smallest computed value of C*j* .

The covariance matrices can be either different for each class or identical. In the first case, we have a quadratic discriminant analysis (QDA) classifier, while in the latter case we have a linear discriminant analysis (LDA) classifier.

### Fisher's Linear Discriminant Analysis

Fisher's linear discriminant analysis (FLDA) is both a projection and classification method. Similar to SVM, we are looking for a linear function *f* (x) = w^*T*^ x + *b* that is used to discriminate multiclass data labels. The method employs adequate dimension reduction from the initial data space to discriminate between *q* classes. The optimization problem becomes finding w defined by *q* − 1 basis vectors.

This technique identifies the first discriminating component based on finding the vector w that maximizes the discrimination index, given as:

(5) $$w^{T}Bw/\left(w^{T}Ww\right)$$

with *B* denoting the interclass sum‐of‐squares matrix and *W* the intraclass sum‐of‐squares matrix.

### Decision Trees

Decision trees represent a nonlinear multistage classifier in which classes are rejected over a sequence of decisions until a finally accepted class is reached. This means that the feature space is split sequentially in specific regions that correspond to the classes. Each feature vector traverses an existing tree based on a sequence of decisions and follows a path of nodes until it reaches the region where it belongs. In other words, the correct class corresponding to a feature vector is determined by searching a tree‐based decision system. This classification scheme is extremely beneficial when a large number of classes is given.

The most popular decision trees are binary decision trees. Binary decision trees separate the search space into hyperrectangles with sides parallel to the axis. The tree is searched in a sequential manner and a decision of the form *xi* ≤ *α*, with *xi* being a feature and *α* a threshold value, is made at each node for individual features.

This processing scheme is an essential part of many tree‐based vector quantization algorithms.

### Fuzzy Classifiers

In crisp classification the membership of each sample from the dataset to a given class is either zero or one. In fuzzy clustering, a sample can belong to more than one class and its membership takes any value between zero and one. A fuzzy classifier is described by the classical fuzzy IF‐THEN rules.^31^, ^32^ A popular algorithm is the fuzzy *c*‐means algorithm, being an unsupervised classification algorithm applied in medical imaging mostly to pixel segmentation. In the beginning, a set of classes has to be determined and the centroid of each class is computed. Each pixel is then classified by its membership values of the classes according to its attributes. Membership value for a certain class indicates the probability of the pixel belonging to that class. The objective of the fuzzy *c*‐means algorithm is to compute membership values to minimize the within‐cluster distances and maximize the between‐cluster distances. The cluster centers are updated iteratively.

### Deep Learning

Classical ML techniques cannot be applied to images directly, and hence it is required to define suitable features (mathematical descriptors) to encode discriminative properties of the lesions of interest. The emergence of deep‐learning (DL) architectures allows working directly with images, and not with extracted or “engineered” features from these images, by learning the feature representation along with the classifiers.^33^

**FIGURE 3:**
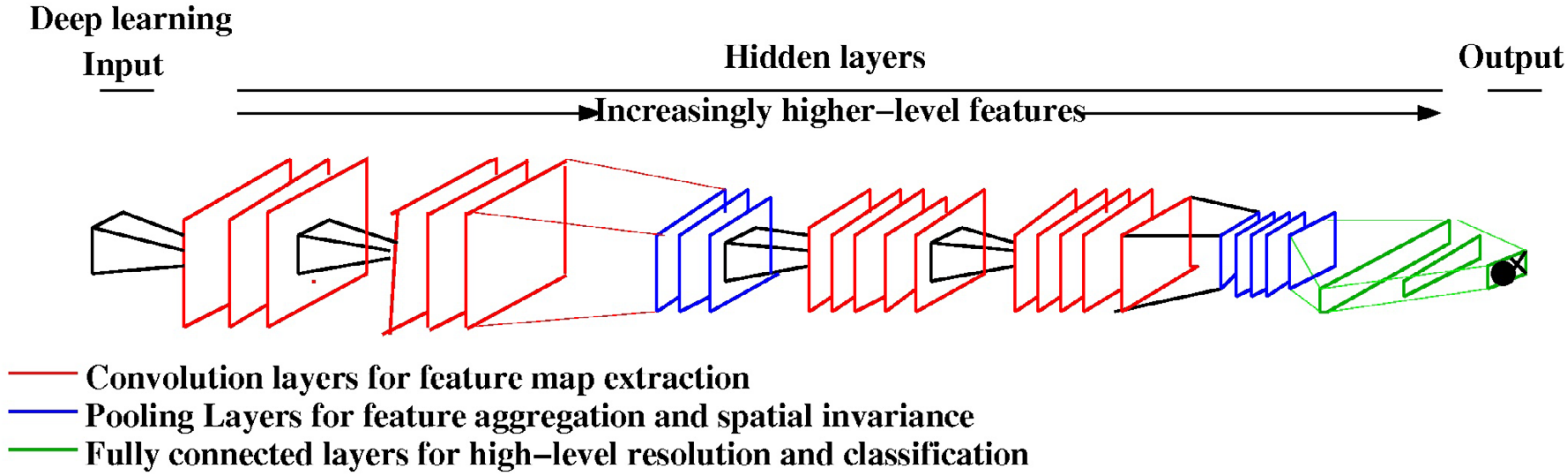
A DL architecture with hidden layers and one output layer.

Inspired from the brain processing in the visual cortex, an architecture achieving several layers of abstraction based on a hierarchy of transformation appears as a well‐suited answer to the above problem. The most common architecture of this type is the convolutional neural network (CNN). Like the human brain, the first layer of this hierarchical network detects edges, then further layers primitive shapes and subsequently more complex visual shapes until a semantics concept is built. The number of layers determines the depth of the network and those networks with up to two hidden layers are considered shallow, while those with more than three are deep. Each layer can be viewed as creating a feature vector while the DL network can be viewed as a modality for learning a hierarchy of features. Thus, the higher layers implement a higher abstraction of the representation or mapping as reflected by the respective feature vector of that layer. With this novel “coding scheme,” the network is generating the features by itself without the need of human intervention. A typical deep CNN is shown in Fig. 3.

Due to the complex nature of DCE‐MRI, with both spatial (volumetric) and temporal variations, feature extraction either through conventional or DL‐based techniques is crucial to achieve good performance. Hundreds of features have been proposed in the literature to encode both morphological (spatial) and kinetics (temporal) properties of the tumor and its enhancement. CNNs were initially proposed to deal with 2D, low‐resolution, RGB images, and therefore need to be adapted in order to effectively process multiparametric inputs and encode both volumetric (spatial) and temporal changes.^34^ At the same time, CNNs are complex networks with millions of parameters that require large datasets for effective training. The combination of these two factors make DL particularly challenging for breast MRI, and especially for lesions such as NMEs, for which collecting large datasets is especially challenging. Indeed, most the reviewed techniques are still firmly based on traditional feature extraction.

### Evaluation Criteria

ML techniques have to be evaluated regarding their performance in the testing phase based on new, previously unseen data samples.

The ability of a classifier to discriminate diseased malignant from benign cases is based on receiver operating characteristic (ROC) analysis. The most important evaluation metrics are accuracy, sensitivity, specificity, and area under the curve (AUC). Accuracy determines the ratio of the correct classified samples in relation to the total samples. The sensitivity (Sn) is the probability that a test result will be positive when the disease is present (true positive rate, expressed as a percentage). The specificity (Sp) is the probability that a test result will be negative when the disease is not present (true negative rate, expressed as a percentage). The AUC represents the degree of separability between the two classes and is a common figure of merit to compare the performance of different classifiers.

## CAD in MRI of the Breast

### CAD in the Detection of Diagnostically Challenging Lesions Based on Tumor‐Extracted Quantitative Features

NME lesions show a heterogeneous appearance in MRI, with high variations in kinetics and morphological characteristics^13^, ^24^, ^35^ and have a lower reported specificity and sensitivity than mass‐enhancing lesions. Most research initiatives in the past have been centered on automated analysis of mass lesions, since they were more straightforward,^9^, ^10^, ^11^, ^36^, ^37^, ^38^, ^39^, ^40^, ^41^, ^42^, ^43^ while very few studies have investigated the characterization of the morphology and/or enhancement kinetic features of NME lesions.^19^, ^44^, ^45^, ^46^ These studies showed a much lower sensitivity and specificity for NME lesions compared with masses, suggesting the need for more advanced algorithms for the diagnosis of nonmass‐like enhancement. The diagnosis of NME lesions is more challenging, as both benign conditions and tumors such as fibrocystic or proliferative changes, and malignant lesions such as ductal carcinoma in situ (DCIS) and invasive lobular cancer (ILC) often present as such.^46^ A systematic classification of NME lesions would be highly beneficial and cost‐effective for clinical management, and would contribute towards a reduction of the number of biopsies and follow‐up exams.

A search through the most important databases was performed to identify various studies related to the employed ML techniques. The primary aim was to categorize the studies according to the following research questions^47^: What are the ML techniques?^48^ What are the evaluation criteria used for their assessment? and^49^ What are the datasets used? Several databases were searched including Springer Link, Web of Science, IEEE Xplore, and PubMed. The following search keywords were used: “breast cancer,” “MR imaging,” “non‐mass lesion,” and “machine learning.”

It is important to provide an improved differential diagnosis for these diagnostically challenging lesions based on a CAD system that ideally incorporates the spatiotemporal properties of these lesions and provides the radiologist with a fast and accurate computational diagnosis support. Available features describe the breast signal in the 4D space and may capture the temporal dynamics, the morphological characteristics, and also the spatial variations within the tumor. In this subsection, techniques that are rooted in quantitative feature extraction are reviewed. Table 1 shows a summary of the articles describing CAD systems for NME lesions based on solely tumor‐extracted morphological features, whereas Table 2 shows a summary of articles describing CAD systems for NME lesions based on both dynamics‐ and tumor‐extracted enhancement curves or spatiotemporal features. The highest predictive value in NME lesions is achieved by both morphological and kinetic parameters.^13^, ^35^A variety of ML techniques have been used for NME analysis, as shown in Tables 1 and 2, with random forest and SVM being the most common choices.

**TABLE 1:** CAD Based on Tumor‐Extracted Morphological Features

ML technique	Performance	Dataset	Reference
Random forest	AUC = 0.9, Acc = 0.88, TP = 0.91, FP = 0.21	106 lesions	^50^
Random forest	Sn = 0.92	50 lesions	^38^
Random forest Naïve Bayes SVM	AUC = 0.74 for RF, AUC = 0.73 for NB, AUC = 0.68 for SVM	162 nonmass lesions	^51^
Quadratic discriminant analysis	AUC = 0.87	84 images	^52^
Random forest	Sn = 0.45, Sp = 0.96	18 lesions	^53^
SVM	AUC = 0.60, Acc = 0.60, Sn = 0.70, Sp = 0.5	46 lesions	^54^
ANN	AUC = 0.76, Sn = 0.87, Sp = 0.56, Acc = 0.81,	54 lesions	^45^
SVM	Sn = 0.87, Sp = 0.56, Acc = 0.81	54 lesions	^55^

**TABLE 2:** CAD Based on Both Dynamics‐ and Tumor‐Extracted Features or Spatiotemporal Features

ML technique	Performance	Dataset	Reference
Random forest	AUC = 0.91, Sn = 0.87, Sp = 0.76,	77 lesions	^56^
SVM	AUC = 0.60, Acc = 0.69, Sn = 0.87, Sp = 0.50	46 lesions	^54^

Based on the reviewed literature, it appears that ML techniques are a promising solution towards NME detection and characterization. There are, however, several challenges to be tackled.First, new techniques are needed for the simultaneous movement correction and segmentation considering spatial and temporal profiles: automatic motion correction represents an important prerequisite for a correct automated lesion evaluation.^57^, ^58^ Therefore, spatial registration has to be performed before enhancement curve analysis. At the same time, accurate segmentation of the lesion is critical, since the spatiotemporal features have to be extracted from the tumor region. Current segmentation algorithms include only spatial properties and are suitable for mass‐enhancing lesions^11^, ^59^, ^60^, ^61^ and will require modifications for NME lesions. Novel elastic combined image registration and segmentation methods based on a variational model and level set approach are needed. These should incorporate spatial as well as temporal contrast‐enhanced images.

Another important point is the development of novel feature extraction for spatiotemporal modeling algorithms that can capture the subtle local variations in NME lesions. BI‐RADS‐based features proved to be insufficient to differentiate between malignant and benign for NME lesions, and therefore additional descriptors are needed to reduce the high proportion of false‐positive diagnosis and unnecessary biopsies.^46^ Automated extracted features that have been applied to lesion characterization capture either variations in their temporal enhancement or in spatial (morphological) structures, or are computed as global features that are unable to capture and describe local variations in the morphological and temporal characteristics of NME lesions. This latter shortcoming can be addressed by implementing novel mathematical spatiotemporal feature descriptors that are able to capture the properties of segmental, focal, dendritic, and clustered ring enhancement.

At the moment, the most known spatiotemporal feature descriptors are: Zernike velocity moments,^62^ the scaling index method,^63^ and voxel‐based adaptive spatiotemporal modeling.^64^, ^65^ Ngo et al showed that spatiotemporal features such as Zernike velocity moments have achieved the highest sensitivity (87.5%) compared with morphologic (62.5%) or kinetic features (70.8%) alone.^54^

The scaling index method is a technique that can capture both morphology and kinetics. Originating from the theory of complex systems, the scaling index extracts the local structure around a given point in an arbitrary dataset. This technique requires converting the image in point distribution, where each voxel corresponds to a point and its state is given by its coordinate and its gray scale intensity value. In the context of MRI of the breast, each point (or voxel) is thus described by its sagittal, coronal, and transverse positions along with the observed intensity value.

In clinical practice, dynamic medical images (ie, images acquired over time) are often assessed qualitatively. However, there is a need to quantify the results from these images in order to provide an objective and effective method for the diagnosis for evaluation of treatment efficacy. Voxel‐based adaptive spatiotemporal modeling can accomplish this. Typically, images suffer from low signal‐to‐noise ratio, which makes quantitative voxelwise evaluation hard. One way to overcome this problem is to aggregate imaging data in an ROI. When using an ROI, however, one obviously loses the spatial information of the image.^65^, ^66^ To this end, a Bayesian approach can be used to gain robust estimates of the voxelwise dynamic.^65^, ^67^ This approach uses the spatial information inherent in the image, to strengthen the local modeling in each voxel. These approaches are usually based on Markov Random Fields (MRF).^68^

### CAD in the Detection of Diagnostically Challenging Lesions Based on Tumor‐Extracted Enhancement Curves

Kinetic parameters extracted either from qualitative BI‐RADS or quantitative empirical mathematical model measures of kinetics have proven not to be useful when it comes to the differential diagnosis of NME lesions.^19^

Table 3 shows a summary of articles describing CAD systems for NME lesions based on tumor‐extracted enhancement curves. The same search criteria were applied as for Table 1.

**TABLE 3:** CAD Based on Tumor‐Extracted Enhancement Curves

ML technique	Performance	Dataset	Reference
SVM	ACC = 0.94, Sn = 0.98, Sp = 0.9, AUC = 0.94	17 images	^69^
Random forest Naïve Bayes SVM	AUC = 0.74 for RF, AUC = 0.73 for NB, AUC = 0.68 for SVM	162 nonmass lesions	^51^
SVM	AUC = 0.77	84 images	^52^
Random forest	Sn = 0.88, Sp = 0.98	18 lesions	^53^
SVM	AUC = 0.65, Sn = 0.65, Sp = 0.75	84 lesions	^70^
SVM	AUC = 0.58, Acc = 0.58, Sn = 0.62, Sp = 0.54,	46 lesions	^54^
ANN	AUC = 0.55, Sn = 0.79, Sp = 0.33, Acc = 0.72	54 lesions	^45^

**FIGURE 4:**
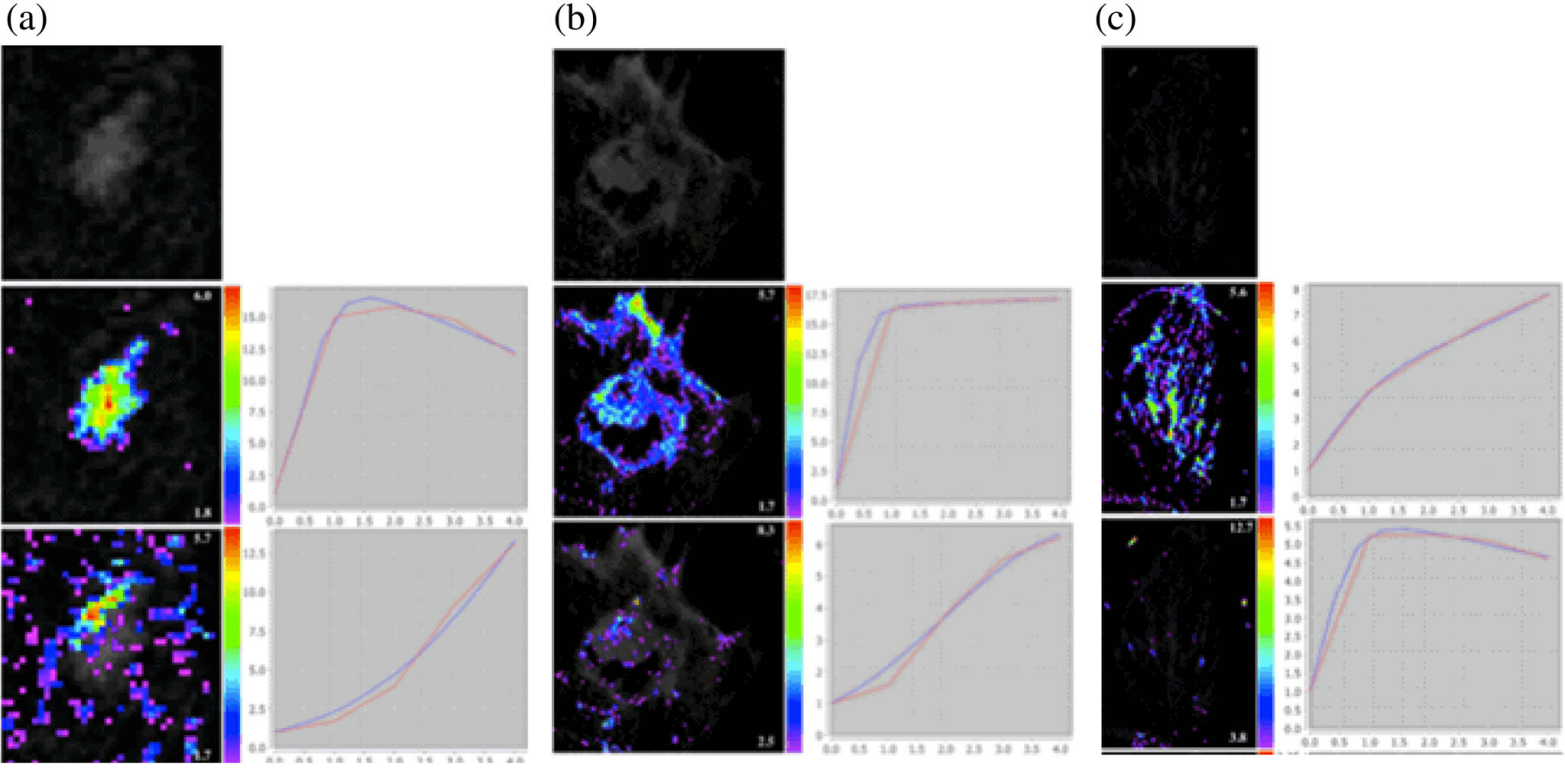
ICA segmentation at 1.5T for a benign (cylindrical cell changes) and two malignant lesions (carcinosarcoma and IDC with surrounding DCIS) showing cluster assignment maps (left of each image) and associated enhancement curves (right of each image) in red and their EMM based on the Gompertzian law in blue.

A simultaneous registration and segmentation can be achieved with independent component analysis (ICA). This technique facilitates the challenging segmentation of NME lesions and does not require a predefined ROI mandatory for manual analysis or an accurate threshold for semiautomated analysis. It incorporates spatial as well as temporal properties and provides an accurate motion correction and segmentation, even for noisy images and spatial smoothness compared with conventional level set methods or clustering‐based techniques. This method detects the interior contours automatically, filters out the noise, and is robust with respect to noise, and includes and evaluates tumor‐specific enhancement curves. ICA has proven to be an excellent method to separate motion artifacts in biomedical image processing^71^, ^72^, ^73^, ^74^, ^75^, ^76^ and recover underlying signals. The task of classifying pixels with similar time‐courses corresponds to finding clusters based on ICA techniques such as topographical ICA^77^ or tree‐dependent component analysis.^78^ Figure 4 shows two cluster assignment maps and the associated time curves for one benign and two malignant lesions. We can see that each lesion has a unique enhancement pattern. Thus, it can be hypothesized that the spatiotemporal behavior of these lesions is determined not by a single ROI‐derived kinetic curve but by two specific interacting signal intensity time curves.

## Future Trends: CAD Systems for Novel Applications

### CAD for Multiparametric MRI

To overcome limitations in specificity, additional functional MRI techniques such as DWI and proton MR spectroscopy have been explored and have demonstrated improved diagnostic accuracy as well as response assessment.^79^ Their combined application is defined as multiparametric MRI for detection and characterization of breast tumors.^13^, ^24^, ^35^, ^80^

Few CAD systems^81^, ^82^, ^83^ for breast masses were proposed for multiparametric MRI. It has been shown^83^ that there is the potential for development of multiparametric CAD that incorporates information from both DWI and DCE‐MRI in breast lesion classification. The multiparametric imaging via MRI / positron emission tomography (PET) and the combination of extracted parameters was shown to improve diagnostic accuracy for breast and prostate lesions ^81^, ^82^ All studies have elucidated that the amount and complexity of the acquired multiparametric data requires the development of advanced analysis tools.

The bottleneck that remains for providing an improved differential diagnosis, and thus contribute to advancing CAD systems beyond the current level, are determining the descriptors that incorporate the diagnostic information from multiparametric MR images for NME lesions. Important steps include:

1. *Development of a novel image normalization framework for these multiparametric images*. The normalization step represents a crucial step for the subsequent feature extraction and classification, since the images stem from heterogeneous sources. Usually, standard preprocessing step is followed by a novel joint segmentation and registration algorithm. A better solution is represented by novel joint segmentation and registration algorithm based on a variational model and level set approach that incorporates spatial as well as temporal contrast‐enhanced images. The multiparametric images are registered such that all segmented images will be in the same reference frame.
  The multiparametric MR images arise from heterogeneous sources and need to be regularized before relevant features for the CAD system can be extracted. This step includes a preprocessing stage and a joint tumor segmentation and registration stage such that all images are in the same reference frame. The preprocessing step includes noise filtering performed based on wavelet shrinkage,^84^ bias correction,^85^ and SI normalization/standardization based on z‐score computation to remove the variability between patients and to enforce the repeatability of the MRI examinations.
  Due to the elasticity and heterogeneity of breast tissue, only nonrigid image registration methods are suitable. At the same time, accurate segmentation of the lesion is critical since the spatiotemporal features have to be extracted from the tumor region. Different solutions have been proposed to solve this problem: these range from purely image‐based statistical and geometrical models for regularization^86^ to more accurate physics‐based models for mechanical deformation^87^ and nonrigid diffeomorphic registration algorithms for volumetric 3D images.^88^, ^89^ The segmentation algorithm is applied to 3D images and uses the information from all available images when determining obscured boundaries, as in the case of NME lesions. This new algorithm can detect the interior contours automatically and provide an accurate motion correction and segmentation even for noisy images and spatial smoothness compared with conventional level set methods or clustering‐based techniques.
2. Identifying novel descriptors such as structure tensors and texture from T_2_‐MRI and advanced DWI methods such as intravoxel incoherent motion (IVIM) maps, restriction spectrum imaging, or multidimensional DWI. The apparent diffusion coefficient (ADC) is the most prevalent method for quantifying diffusion in clinical practice and is based on fitting a monoexponential model usually to two images acquired without diffusion‐weighting and with relatively high diffusion‐weighting. However, lesion heterogeneity is insufficiently described by a single ADC threshold and thus more detailed structural and functional image features have to be extracted from T_2_‐MRI and DWI. Novel descriptors should include additional information from multiparametric MRI and capture the structure of the breast tissue in a unique manner. Experimentally, the monoexponential fit provided by the ADC was in practice found to be only applicable to simple cysts, whereas malignant and benign lesions required a more complex biexponential model fitted from six or more images with varying diffusion‐weighting parameters.^90^ Techniques such as IVIM provide separatequantitative parameters for tissue diffusivity, perfusion fraction, and pseudodiffusion and has been shown to be helpful for the differentiation between benign and malignant breast lesions.^90^ This provides motivation for further research regarding the suitability of the IVIM features in DWI for nonmass‐enhancing lesions.
  A few studies have exploited first‐order texture measurements statistics reflecting the lesion ADC heterogeneity,^91^ an approach that has already demonstrated increased potential in MRI for prostate cancer. Nineteen different texture features were extracted describing the image from the gray‐level co‐occurrence matrix (GLCM): contrast, correlation, cluster prominence, cluster shade, dissimilarity, energy, entropy, homogeneity, maximum probability, sum of squares, sum average, sum variance, sum entropy, difference variance, difference entropy, information measure of correlation, inverse difference, inverse difference normalized, and inverse difference moment normalized. These features have the potential to characterize homogeneity, gray‐level transitions, and the presence of organized structures.
3. *Identifying novel spatiotemporal descriptors from DCE‐MRI images as the most powerful discriminators*. Some lesions exhibit a high variance in morphological and kinetic characteristics and the consequence is a high proportion of false‐positive diagnoses.^46^ Automated extracted features that have been applied to lesion characterization are either features that capture the variations in their temporal enhancement or in spatial (morphological) structures or are global features that are unable to describe local information. To address this latter shortcoming, novel mathematical spatiotemporal feature descriptors are needed such as local velocity moments, scaling index, and dynamic texture derived from geometrical multiscale decomposition that are able to capture the segmental, focal, linear, regional, and diffuse, and internal enhancement patterns (homogeneous, heterogeneous, clumped, clustered ring enhancement, dendritic), and lesion heterogeneity.

Dynamic texture features can be extracted based on the 2D+T curvelet transform.^92^ It yields a spatiotemporal decomposition that represents an extension of the temporal domain of the 2D curvelet transform.

This novel technique is relevant for extracting nonlocal phenomena propagating temporally and operates based on a geometrical multiscale decomposition. As in the 2D case, a separable 3D convolution can be factored into 1D convolution along rows, columns, and image indexes of the MRI scans. As a result of this transform, a spatiotemporal segmentation algorithm is produced. The coefficients of the 2D+T curvelet transform contain discriminative information that can be employed for recognizing different dynamic textures. As in the case of 2D texture, the wavelet decomposition is employed to build feature vectors from detail subbands. The feature vector is composed by the average, standard deviation, energy, and entropy of the detail subbands. By adding a discrete cosine transformation to the 2D+T curvelet transform a morphological transformation can be implemented that considers also the local phenomena. Thus, the geometry of the dynamic texture can be additionally captured. This novel descriptorcould sufficiently represent the dynamical properties of the temporal texture characterizing the heterogeneous behavior of diagnostically challenging lesions.

**FIGURE 5:**
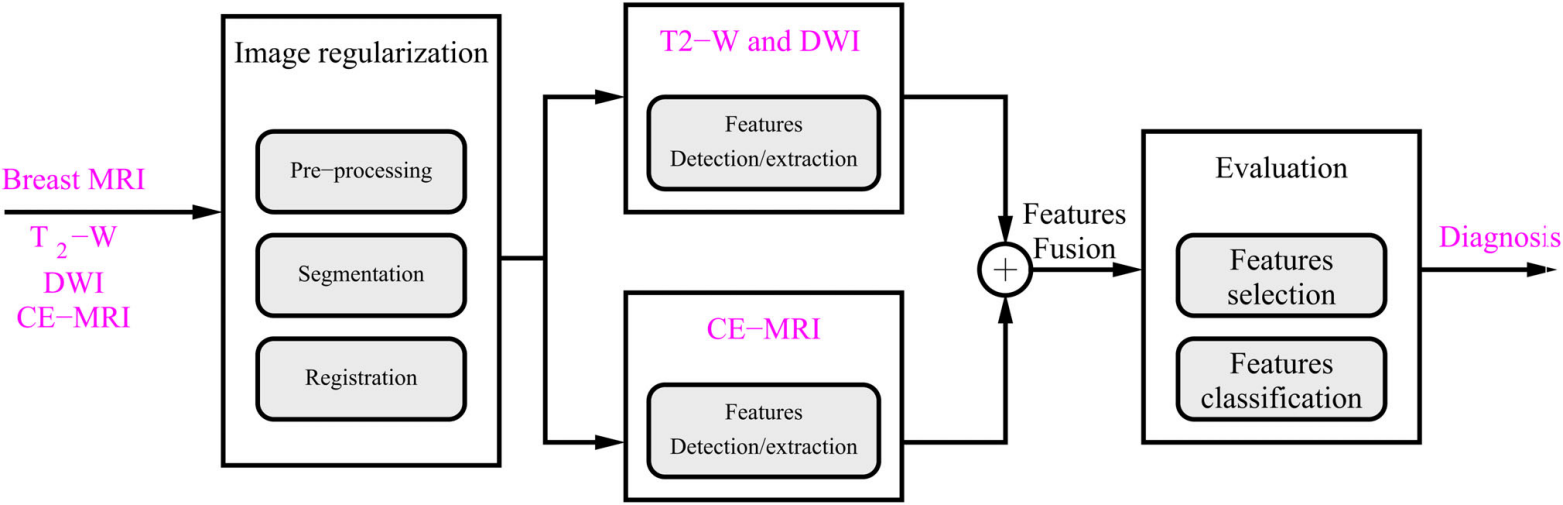
CAD system for multiparametric breast MR images.

A possible CAD system for multiparametric breast MR images is shown in Fig. 5. Key components should be: spatiotemporal descriptors and tensor fields for the evaluation of diagnostically challenging lesions from multiparametric 3T images, thus increasing specificity without compromising the sensitivity of DCE‐MRI.

### CAD in Neoadjuvant Chemotherapy

Neoadjuvant chemotherapy (NAC) is the standard of care and is widely used in patients with locally advanced breast cancer, offering several advantages, such as reduction of tumor and enabling breast‐conservation surgery instead of mastectomy as well as response‐guided NAC approaches. In patients undergoing NAC for breast cancer the achievement of a pathological complete response (pCR) is associated with a significantly improved disease‐free and overall survival. However, a pCR is achieved in only 30% of the patients after the completion of NAC and clinical studies have shown that the therapeutic outcome can be improved after treatment modifications during NAC. Predicting the pathological response after NAC in breast cancer patients is crucial and quantitative computerized methods represent an important step towards an accurate and effective breast cancer treatment. The first study assessing the role of an automatic CAD system in DCE‐MRI predicting the pathological response to NAC has been described.^93^

**FIGURE 6:**
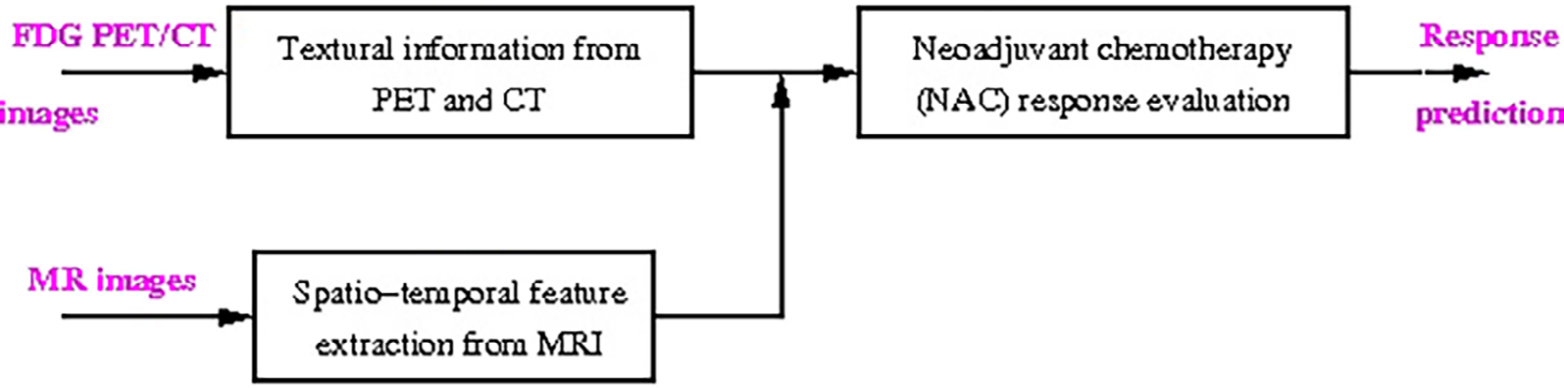
CAD system for NAC.

Tumor response is monitored in the latest clinical studies with PET/MRI. These techniques vary a lot in the performance of NAC response monitoring of different breast cancer types and the combined use of PET and MRI has been shown to have a complementary value^94^, ^95^; however, there is still room for improvement. The success of therapeutics in breast cancer could be improved based on developing novel distinctive and consistent imaging parameters extracted from a combined use of PET and MRI that are tailored for enhancing the pCR after NAC and validating them in a CAD scheme. Such a possible CAD scheme is shown in Fig. 6.

Several ML techniques were applied for NAC, mostly for breast masses. A CAD scheme based on a Bayesian classifier^93^ used DCE‐MRI data and extracted texture features from an automatically segmented 3D mask of the tumor and predicted pathological response to NAC. A similar method based on radiomics was employed.^96^ A Gaussian SVM processing quantitative kinetic and texture‐based image features from MR images for NAC has been proposed.^48^ An SVM was also applied for NAC.^97^, ^98^ An ANN processes a new clinical marker based on quantitative kinetic image features analysis and assessing its feasibility for NAC was presented.^49^ Fuzzy c‐means clustering was employed for NAC in connection with level set segmentation.^60^

DL methods have been applied to automatically score HER2, a biomarker that determines the patients who are eligible for anti‐HER2 targeted therapies.^99^ That study shows that DL is able to identify cases that are most likely misdiagnosed in the traditional clinical decision‐making. An important application of DL applied to NAC when analyzing different contrast timepoints has been shown^100^: they applied CNNs to extract features from DCE‐MRI and determined that the image acquired before contrast injection was the most effective at predicting response to therapy, with performance moderately increasing when including also images acquired after contrast injection.

### Breast Cancer Radiomics

In the past 3 years, a novel computational approach—radiomics—is emerging to represent oncological tissues based on quantitative descriptors.^47^ Currently, in computational radiology there are two concurrent research lines: radiomics and artificial intelligence (AI). Radiomics is the ML‐based approach of extracting handcrafted features descriptive of a tumor, while AI employs DL techniques and works directly with the medical images.

Radiomics represents a novel approach to achieve a detailed quantification of the tumor phenotypes by analyzing a large number of image descriptors. It has been hypothesized that a large number of radiomic features tremendously increase the diagnostic, prognostic, and predictive power. With the increasing importance of “personalized medicine,” new treatment strategies are being sought to respond to the specific characteristics of each patient and cancer phenotype. So far, personalized medicine is centered around molecular characteristics with genomics and proteomics data analysis. In Hoffman et al,^101^ a quantitative radiomics approach was applied based on shape, texture, and kinetics tumor features and was evaluated in comparison with a reduced‐order feature approach in a CAD system applied to diagnostically challenging lesions.

The potential of radiomics as a training‐independent diagnostic decision tool has been shown.^102^ The radiomics classifiers performed well in the differentiation of malignant and benign lesion; however, their performance was lower than that of an experienced radiologist. Prasanna et al introduced a new radiomics descriptor, the Co‐occurrence of Local Anisotropic Gradient Orientations (CoLlAGe).^103^ It is able to distinguish benign and pathologic phenotypes when they appear similar to each other on anatomic imaging. This new descriptor can capture their local entropy patterns and thus reflect hidden local differences in the tissue microarchitecture.

A comparison between DL and radiomics was performed^104^, ^105^ and a fusion between DL and CNN‐extracted features.^106^ The benefit of including multiple radiomic features, automatically extracted, in a lesion signature significantly improved the ability to distinguish between benign lesions and luminal A breast cancers, compared to using maximum linear size alone.^107^ The diagnostic accuracy was evaluated^108^ using ROI‐based, radiomics, and DL methods, by taking peritumor tissue into consideration. A few studies are employing the radiomics approach in connection with multiparametric breast images for NAC prediction.^90^, ^109^ The only large study including NME lesions was presented in Ref. 110. The specifics of this study are described in Table 4.

**TABLE 4:** CAD Based on Tumor Radiomics‐Extracted Features

ML technique	Evaluation results	Used dataset	References
SVM	AUC = 0.9	509 patients	^110^

### Standardization and Repeatibility in Breast DCE‐MRI

Advanced breast imaging techniques such as DCE‐MRI and DWI are complex and highly adjustable procedures. The difference in hardware and software implemented by different vendors can produce noticeable differences in image quality and appearance. In addition, acquisition protocols vary across and within studies, vendors, and acquisition centers, and may include different spatiotemporal resolutions, contrast agents, or imaging parameters (TR, TE, fat suppression, etc.). Postprocessing, including delineation and segmentation of the tumoral area, may further complicate this picture. ML models rely on quantitative features, either hand‐engineered or learned by CNNs, which may be heavily affected by such changes. Since collecting data for all possible acquisition protocols is unfeasible, these aspects need to be carefully considered in the design, training, and validation of ML models. The problem of how to design robust ML models that can generalize to multiple settings is still, in many ways, an open research question.

There are two main approaches in order to build ML models robust to acquisition parameters: image standardization/harmonization and more robust feature extraction/selection. Besides working alongside vendors to standardize image acquisition, a laudable but notoriously difficult quest, image or feature harmonization may be more feasible. Feature harmonization was demonstrated to significantly improve benign vs. malignant lesion classification in an DCE‐MRI dataset acquired from multiple international institutions.^111^ The harmonization was applied separately within features categories, that is, morphology, texture, and kinetics, by aligning the distribution of features from multiple centers, after adjusting for covariates. Still, a large dataset including more than 1000 cancer cases per institution was available, which may be unfeasible to collect for lesion subtypes such as NME.

Another line of research analyzes repeatability and reproducibility of individual features in order to select those features that guarantee a higher reproducibility.^112^ In a systematic literature review published in 2018, this aspect was extensively investigated for imaging modalities such as CT and PET, whereas only study was available for MRI.^112^ Indeed, MRI involves larger variability in imaging parameters and requires extending the analysis to temporal as well as spatial features. A recent study analyzed the effect of acquisition parameters (specifically, scanner model, magnetic field strength, and slice thickness) on features related to lesion and fibroglandular tissue morphology, texture, and enhancement.^113^ The authors found that these features have a significant effect on the extracted radiomics/radiogenomic features; however, those extracted from fibroglandular tissue are more susceptible to image parameters than those extracted from the tumor area, which is encouraging, as the latter are of higher clinical interest. However, more studies are needed to cover a wider range of imaging parameters and features. Another important issue to be settled is whether CNN‐based features are more robust than hand‐engineered features to such variations.

## Discussion

This systematic review aimed to give an overview of the currently available methodology and applications of ML‐based CAD systems for diagnostically challenging lesions in MRI of the breast. ML techniques have been successfully applied in medical image processing. Over the past decades, we have witnessed the transition of ML techniques from feature extraction from medical images to working directly with the raw images, as enabled by newer models such as CNNs.

To date, applications of state‐of‐the‐art CAD systems are based on established feature engineering and enhancement curves extraction from DCE‐MRI; these techniques have proven to be valuable tools for the detection and diagnosis in clinical praxis. Radiologists can benefit from such ML‐based CAD systems, resulting in reduced interobserver variability and improved interpretation of breast imaging for the presence or absence of breast cancer.

Future directions for research and development aim to develop ML‐based CAD systems not only for diagnostic but also predictive and prognostic purposes, by including other MRI methods such as T_2_‐weighted or DW sequences or hybrid (PET/MRI) techniques, as well as extracted quantitative radiomics features. Such advanced multiparametric ML‐based CAD systems are expected to further improve not only diagnostic accuracy for challenging lesions but also provide predictive and prognostic indicators for breast cancer. It has to be noted that, despite encouraging results, we are still at the dawn of a widespread implementation of ML‐based CAD systems in breast MRI. To date, studies have been mainly retrospective, single‐institution, using different equipment, scan protocols, sequence parameters, and postprocessing steps, and have included relatively small numbers of patients, which limits the statistical power of the studies and may compromise the generalizability of the results. Rigorous standardization of MRI hardware and software, quantitative MRI techniques, and multicenter large‐scale studies are needed to build and validate robust machine‐learning models that are applicable across patients and institutions to provide clinical value.

## References

[1] Leithner D, Wengert G, Helbich T, et al. Clinical role of breast MRI now and going forward. Clin Radiol 2017;73:700‐714. 29229179 10.1016/j.crad.2017.10.021 PMC6788454

[2] Mann R, Moes R, van Zelst J, Geppert C, Karssemeijer N, Platel B. A novel approach to contrast‐enhanced breast magnetic resonance imaging for screening high‐resolution ultrafast dynamic imaging. Investig Radiol 2014;49:579‐585. 24691143 10.1097/RLI.0000000000000057

[3] Mann R, Balleyguier C, Baltzer P, et al. Breast MRI: EUSOBI recommendations for women information. Eur Radiol 2015;25:3669‐3678. 26002130 10.1007/s00330-015-3807-z PMC4636525

[4] Marino M, Helbich T, Baltzer P, Pinker‐Domenig K. Multiparametric MRI of the breast: A review. J Magn Reson Imaging 2018;47:301‐315. 28639300 10.1002/jmri.25790

[5] Heywang S, Wolf A, Pruss E. MRI imaging of the breast: Fast imaging sequences with and without GD‐DTPA. Radiology 1989;171:95‐103. 2648479 10.1148/radiology.171.1.2648479

[6] Yousef E, Duchesneau R, Alfidi R. Magnetic resonance imaging of the breast. Radiology 1984;150:761‐766. 6695077 10.1148/radiology.150.3.6695077

[7] Lucht E, Delorme S, Brix G. Neural network‐based segmentation of dynamic (MR) mammography images. Magn Reson Imaging 2002;20:89‐94. 10.1016/s0730-725x(02)00464-2 12034335

[8] Arbash Meinel L, Stolpen A, Berbaum K, Fajardo L, Reinhardt J. Breast MRI lesion classification: Improved performance of human readers with a backpropagation network computer‐aided diagnosis (CAD) system. J Magn Resonan Imaging 2007;25:89‐95. 10.1002/jmri.20794 17154399

[9] Szabo B, Wilberg M, Bone B, Aspelin P. Application of artificial neural networks to the analysis of dynamic MR imaging features to the breast. Eur Radiol 2004;14:1217‐1225. 15034745 10.1007/s00330-004-2280-x

[10] Ertas G, Gulcur O, Osman O, Ucan O, Tunaci M, Dursun M. Breast MR segmentation and lesion detection with cellular neural networks and 3D template matching. Comput Biol Med 2008;38:116‐126. 17854795 10.1016/j.compbiomed.2007.08.001

[11] Chen W, Giger M, Newstead G, Bick U. Automatic identification and classification of characteristic kinetic curves of breast lesions on DCE‐MRI. Med Phys 2006;33:2878‐2887. 16964864 10.1118/1.2210568

[12] Stoutjesdijk M, Veltman J, Huisman H, et al. Automated analysis of contrast enhancement in breast MRI lesions using mean shift clustering for ROI selection. J Magn Reson Imaging 2007;26:606‐614. 17729367 10.1002/jmri.21026

[13] Yabuuchi H, Matsuo Y, Kamitani T, et al. Non‐mass‐like enhancement on contrast‐enhanced breast MRI imaging: Lesion characterization using combination of dynamic contrast‐enhanced and diffusion‐weighted MR images. Eur J Radiol 2010;75:126‐132. 10.1016/j.ejrad.2009.09.013 19796900

[14] Kuhl CK, Mielcareck P, Klaschik S, et al. Dynamic breast MR imaging: Are signal intensity time course data useful for differential diagnosis of enhancing lesions? Radiology 1999;211:101‐110. 10189459 10.1148/radiology.211.1.r99ap38101

[15] Laub G, Kroecker R. Syngo twist for dynamic time‐resolved MR angiography. Magn Flash 2006;3:92‐95.

[16] Saranathan M, Rettmann DW, Hargreaves B, Clarke S, Vasanawala S. Differential subsampling with cartesian ordering (disco): A high spatio‐temporal resolution Dixon imaging sequence for multiphasic contrast enhanced abdominal imaging. Magn Reson Imaging 2012;35:1484‐1492. 10.1002/jmri.23602 PMC3354015 22334505

[17] Tudorica L, Oh K, Roy N, et al. A feasible high spatiotemporal resolution breast DCE‐MRI protocol for clinical settings. Magn Reson Imaging 2012;30:1257‐1267. 22770687 10.1016/j.mri.2012.04.009 PMC3466402

[18] Platel B, Mus R, Welte T, Karssemeijer N, Mann R. Automated characterization of breast lesions imaged with an ultrafast DCE‐MR protocol. IEEE Trans Med Imaging 2014;33(2):225‐232. 24058020 10.1109/TMI.2013.2281984

[19] Jansen SA, Shimauchi A, Zak L, Fan X, Karczmar GS, Newstaed GM. The diverse pathology and kinetics of mass, nonmass, and focus enhancement on MR imaging of the breast. J Magn Reson Imaging 2011;33:1382‐1389. 21591007 10.1002/jmri.22567 PMC3098464

[20] Schnall MD, Rosten S, Englander S, Orel S, Nunes L. A combined architectural and kinetic interpretation model for breast MR images. Acad Radiol 2001;8:591‐597. 11450959 10.1016/S1076-6332(03)80683-9

[21] Stoutjesdijk M, Fuetterer J, Boetes C, van Dienand L, Jaeger G, Barentsz J. Variability in the description of morphologic and contrast enhancement characteristics of breast lesions on magnetic resonance imaging. Investig Radiol 2005;40:355‐362. 15905722 10.1097/01.rli.0000163741.16718.3e

[22] Agliozzo S, De Luca M, Bracco C, et al. Computer‐aided diagnois for contrast‐enhanced breast MRI of mass‐like lesions using a multi‐parametric model combining a selection of morphological, kinetic and spatio‐temporal features. Med Phys 2012;39:3102‐3109. 22482596 10.1118/1.3691178

[23] Szabo B, Aspelin P, Wiberg M, Bone B. Dynamic MR imaging of the breast—Analysis of kinetic and morphologic diagnsotic criteria. Acta Radiol 2003;44:379‐386. 12846687 10.1080/j.1600-0455.2003.00084.x

[24] Rosen E, Smith‐Foley S, DeMartini W, Eby P, Peacock S, Lehman C. Bi‐RADS MRI enhancement characteristics of ductal carcinoma in situ. Breast J 2007;13:545‐550. 17983393 10.1111/j.1524-4741.2007.00513.x

[25] Erguvan‐Dogan B, Whitman GJ, Kushwaha AC, Phelps MJ, Dempsey PJ. Bi‐RADS‐MRI: a primer. Am J Roentgenol 2006;187(2):W152‐W160. 16861504 10.2214/AJR.05.0572

[26] Breiman L. Random forests. Mach Learn 2001;45(1):5‐32.

[27] Cristiani N, Shawe‐Taylor J. An introduction to support vector machines and other kernel‐based learning methods. Cambridge, MA: Cambridge University Press; 2000.

[28] Schölkopf B. Support vector learning. Munich: R. Oldenbourg Verlag; 1997.

[29] Vapnik VN. The nature of statistical learning theory. New York: Springer; 2000.

[30] Theodoridis S, Koutroumbas K. Pattern recognition. Cambridge, MA: Academic Press; 1998.

[31] Baraldi A, Blonda P. A survey of fuzzy clustering algorithms for pattern recognition—Part I. IEEE Trans Syst Man Cybern 1999;29:778‐785. 10.1109/3477.809032 18252357

[32] Baraldi A, Blonda P. A survey of fuzzy clustering algorithms for pattern recognition—Part II. IEEE Trans Syst Man Cybern 1999;29:786‐801. 10.1109/3477.809033 18252358

[33] LeCun Y, Bengio Y, Hinton G. Deep learning. Nature 2015;521:436‐444. 26017442 10.1038/nature14539

[34] Morra L, Delsanto S, Correale L. Artificial intelligence in medical imaging: From theory to clinical practice. Boca Raton, FL: CRC Press; 2019.

[35] Sakamoto N, Tozaki M, Higa K, et al. Categorization of non‐mass‐like breast lesions detected by MRI. Breast Cancer 2008;15:241‐246. 18224381 10.1007/s12282-007-0028-6

[36] Dalmis M, Gubern‐Merida A, Vreemann S, et al. Artificial intelligence‐based classification of breast lesions imaged with a multiparametric breast MRI protocol with ultrafast DCE‐MRI, T2, and DWI. Investig Radiol 2019;54:325‐332. 30652985 10.1097/RLI.0000000000000544

[37] Gao F, Wu T, Li J, et al. Sd‐CNN: A shallow‐deep CNN for improved breast cancer diagnosis. Comput Med Imaging Graph 2018;70:53‐62. 30292910 10.1016/j.compmedimag.2018.09.004

[38] Gubern‐Merida A, Marti R, Melendez J, et al. Automated localization of breast cancer in DCE‐MRI. Med Image Anal 2015;20:265‐274. 25532510 10.1016/j.media.2014.12.001

[39] Hadad O, Bakalo R, Hashoul S, Amit G. Classification of breast lesions using cross‐modal deep learning. ISBI 2017;1:109‐112.

[40] Lucht R, Delorme S, Heiss J, et al. Classification of signal‐time curves obtained by dynamic‐magnetic resonance mammography. Investig Radiol 2005;40:442‐447. 15973136 10.1097/01.rli.0000164788.73298.ae

[41] Meyer‐Bäse A, Schlossbauer T, Lange O, Wismüller A. Small lesions evaluation based on unsupervised cluster analysis of signal‐intensity time courses in dynamic breast MRI. Int J Biomed Imaging 2010;326924. 10.1155/2009/326924 PMC2850138 20379361

[42] Zheng H, Gu Y, Qin Y, Huang X, Yang J, Yang GZ. Small lesion classification in dynamic contrast enhancement MRI for breast cancer early detection. MICCAI 2018;1:876‐884.

[43] Zhu J, Alwadawy E, Saha A, Zhang Z, Harowicz H, Mazurowski M. Deep learning for identifying radiogenomic associations in breast cancer. Comput Biol Med 2019;109:85‐90. 31048129 10.1016/j.compbiomed.2019.04.018 PMC7155381

[44] Jansen S. Ductal carcinoma in situ: Detection, diagnosis, and characterization with magnetic resonance imaging. Semin Ultrasound CT MRI 2011;32:306‐318. 10.1053/j.sult.2011.02.007 21782121

[45] Newell D, Nie K, Chen J, et al. Selection of diagnostic features on breast MRI to differentiate between malignant and benign lesions using computer‐aided diagnostics: Differences in lesions presenting as mass and non‐mass‐like enhancement. Eur Radiol 2010;20:771‐781. 19789878 10.1007/s00330-009-1616-y PMC2835636

[46] Vag T, Baltzer P, Dietzel M, et al. Intravoxel incoherent motion (IVIM) in evaluation of breast lesions: Comparison with conventional DWI. Eur J Radiol 2013;21:782‐789. 10.1016/j.ejrad.2013.08.006 24034833

[47] Aerts H, Velazquez E, Leijenaar R, et al. Decoding tumour phenotype by noninvasive imaging using a quantitative radiomics approach. Nat Commun 2014;5:4644. 10.1038/ncomms5006 PMC4059926 24892406

[48] Aghaei F, Hollingsworth AB, Mirniaharikandehei S, Wang Y, Liu H, Zheng B. Developing a new quantitative imaging marker to predict pathological complete response to neoadjuvant chemotherapy. SPIE Med Imaging 2019;10950:UNSP109502O.

[49] Aghaei F, Tan M, Hollingsworth AB, Qian W, Liu H, Zheng B. Computer‐aided breast MR image feature analysis for prediction of tumor response to chemotherapy. Med Phys 2015;42:6520‐6528. 26520742 10.1118/1.4933198 PMC4617733

[50] Razavi M, Wang L, Tan T, et al. Novel morphological features for non‐mass‐like breast lesion classification on DCE‐MRI. Mach Learn Med Imaging 2016;10019:305‐312.

[51] Wang L, Harz M, Boehler T, Platel B, Homeyer A, Hahn HK. A robust and extendable framework towards fully automated diagnosis of nonmass lesions in breast DCE‐MRI. ISBI 2014;1:129‐132.

[52] Hoffmann S, Shutler JD, Lobbes M, Burgeth B, Meyer‐Baese A. Automated analysis of diagnostically challenging lesions in breast MRI based on spatio‐temporal moments and joint segmentation‐motion compensation technique. EURASIP J Adv Signal Process 2013;2013:172.

[53] Srikantha A, Harz MT, Newstead G, et al. Symmetry‐based detection and diagnosis of DCIS in breast MRI. SPIE Med Imaging 2013;8670:86701E.

[54] Ngo D, Lobbes M, Lockwood M, Meyer‐Baese A. Spatio‐temporal feature extraction for differentiation of non‐mass‐enhancing lesions in breast MRI. SPIE Symp Comput Intellig 2012;8367:8367‐8369.

[55] Keyvanfard F, Shoorehdeli MA, Teshnehlab M, Nie amd K, Su MY. A robust and extendable framework towards fully automated diagnosis of nonmass lesions in breast DCE‐MRI. Neural Comput Appl 2013;22:S35‐S45.

[56] Gallego‐Ortiz C, Martel AL. Classification of breast lesions presenting as mass and non‐mass lesions. SPIE Med Imaging 2014;9035:90351Z.

[57] Behrens S, Laue H, Boehler T, Kuemmerlen B, Hahn H, Peitgen HO. Computer assistance for MR based diagnosis of breast cancer: Present and future challenges. Comput Med Imaging Graph 2007;31:236‐247. 17369019 10.1016/j.compmedimag.2007.02.007

[58] Hill A, Mehnert A, Crozier S, McMahon K. Evaluating the accuracy and impact of registration in dynamic contrast‐enhanced breast MRI. Concepts Magn Reson B 2009;35B:106‐120.

[59] Arbash Meinel L, Buelow T, Huo D, et al. Robust segmentation of mass‐lesions in contrast‐enhanced dynamic breast MR images. J Magn Reson Imaging 2010;32:110‐119. 20578017 10.1002/jmri.22206

[60] Shi J, Sahiner B, Chan H, et al. Treatment response assessment of breast masses on dynamic contrast‐enhanced magnetic resonance scans using fuzzy c‐means clustering and level set segmentation. Med Phys 2009;36:5052‐5063. 19994516 10.1118/1.3238101 PMC2773457

[61] Zheng Y, Englander S, Baloch S, et al. Step: Spatiotemporal enhancement pattern for MR‐based breast tumor diagnosis. Med Phys 2009;36:3192‐3204. 19673218 10.1118/1.3151811 PMC2852449

[62] Shutler JD, Nixon MS. Zernike velocity moments for sequence‐based description of moving features. Image Vis Comput 2006;24:343‐356.

[63] Jamitzky F, Stark R, Bunk W, et al. Scaling‐index method as an image processing tool in scanning‐probe microscopy. Ultramicroscopy 2001;86:241‐246. 11215629 10.1016/s0304-3991(00)00111-x

[64] Schmid VJ. Voxel‐based adaptive spatio‐temporal modelling of perfusion cardiovascular MRI. IEEE Trans Med Imaging 2011;30(7):1305‐1313. 21296707 10.1109/TMI.2011.2109733

[65] Schmid VJ, Whitcher B, Padhani AR, Taylor NJ, Yang G‐Z. Bayesian methods for pharmacokinetic models in dynamic contrast‐enhanced magnetic resonance imaging. IEEE Trans Med Imaging 2006;25(12):1627‐1636. 17167997 10.1109/tmi.2006.884210

[66] Kelm BM, Menze BH, Nix O, Zechmann CM, Hamprecht FA. Estimating kinetic parameter maps from dynamic contrast‐enhanced MRI using spatial prior knowledge. IEEE Trans Med Imaging 2009;28(10):1534‐1547. 19369150 10.1109/TMI.2009.2019957

[67] Orton MR, Collins DJ, Walker‐Samuel S, et al. Bayesian estimation of pharmacokinetic parameters for DCE‐MRI with a robust treatment of enhancement onset time. Phys Med Biol 2007;52(9):2393‐2408. 17440242 10.1088/0031-9155/52/9/005

[68] Rue H, Held L. Gaussian Markov random fields: Theory and applications (monographs on statistics and applied probability). London: Chapman & Hall; 2005.

[69] Illan IA, Tahmassebi A, Meyer‐Baese A. Machine learning for accurate differentiation of benign and malignant breast tumors presenting as non‐mass enhancement. In: Proc SPIE 10669, Computational Imaging III; 2018.

[70] Goebl S, Plant C, Lobbes M, Meyer‐Baese A. CAD‐system based on kinetic analysis for non‐mass‐enhancing lesions in DCE‐MRI. SPIE Symp Comput Intellig 2013;8750:87500R.

[71] Calhoun V, Adali T. Unmixing functional magnetic resonance imaging with independent component analysis. IEEE Eng Med Biol 2006;25:79‐90. 10.1109/memb.2006.1607672 16568940

[72] Calhoun V, Adali T, Pearlson G, Pekar J. Spatial and temporal independent component analysis of functional MRI data containing a pair of task–related waveforms. Hum Brain Mapping 2001;13:43‐53. 10.1002/hbm.1024 PMC6871956 11284046

[73] Calhoun V, Pekar J, McGinty V. Different activation dynamics in multiple neural systems during simulated driving. Hum Brain Mapping 2002;16:158‐167. 10.1002/hbm.10032 PMC6872105 12112769

[74] Meyer‐Bäse A, Wismueller A, Lange O. Comparison of two exploratory data analysis methods for fMRI: Unsupervised clustering versus independent component analysis. IEEE Trans Inform Technol Biomed 2004;8:387‐398. 10.1109/titb.2004.834406 15484444

[75] Meyer‐Bäse A, Wismueller A, Lange O, Auer D, Sumners D. Model‐free fMRI analsyis using topographic independent component analysis. Int J Neural Syst 2004;14:217‐228. 15372699 10.1142/S0129065704002017

[76] Saalbach A, Lange O, Nattkemper T, Meyer‐Bäse A. On the application of (topographic) independent and tree‐dependent component analysis for the examination of DCE‐MRI data. Biomed Signal Process Control 2009;4:247‐253. 20689662 10.1016/j.bspc.2009.03.010 PMC2916199

[77] Hyvarinen A, Hoyer P. Topographic independent component analysis. Neural Comput 2001;13:1527‐1558. 11440596 10.1162/089976601750264992

[78] Bach FR, Jordan MI. Beyond independent components: Trees and clusters. J Mach Learn Res 2003;4:1205‐1233.

[79] Pinker K, Moy L, Sutton EJ, et al. Diffusion‐weighted imaging with apparent diffusion coefficient mapping for breast cancer detection as a stand‐alone parameter: Comparison with dynamic contrast‐enhanced and multiparametric magnetic resonance imaging. Investig Radiol 2018;53:587‐595. 29620604 10.1097/RLI.0000000000000465 PMC6123254

[80] Jacobs MA, Barker PB, Bluemke DA, et al. Benign and malignant breast lesions: Diagnosis with multiparametric MR imaging. Radiology 2003;229:225‐232. 14519877 10.1148/radiol.2291020333

[81] Bhooshan N, Giger M, Lan L, et al. Combined use of t‐2‐weighted MRI and t‐1‐weighted dynamic contrast‐enhanced MRI in the automated analysis of breast lesions. Magn Reson Imaging 2011;66:555‐563. 10.1002/mrm.22800 PMC4156840 21523818

[82] Gatidis S, Schmid H, Claussen CD, Schwenzer NF. Multiparametric imaging with simultaneousMRI/pet. Methodological aspects and possible clinical applications. Zeitschrift für Rheumatologie 2015;74:878‐885. 26589201 10.1007/s00393-015-0011-0

[83] Hu Q, Whitney HM, Edwards A, Papaioannou J, Giger ML. Radiomics and deep learning of diffusion‐weighted MRI in the diagnosis of breast cancer. SPIE Med Imaging 2019;10950:UNSP109504A.

[84] Ampeliotis D, Anonakoudi A, Berberidis K, Psarakias E. Computer aided detection of prostate cancer using fused information from dynamic contrast enhanced and morphological magnetic resonance imaging. IEEE Int Conf Signal Process Commun 2007;2:888‐891.

[85] Madabhushi A, Udapa J. New methods for of MR image intensity standardization via generalized scale. Comput Vis Image Understand 2006;101:100‐121. 10.1118/1.2335487 17022239

[86] Wang Y, Staib L. Physical model‐based non‐rigid registration incorporating statistical shape information. Med Image Anal 2000;4:7‐20. 10972317 10.1016/s1361-8415(00)00004-9

[87] Schnabel J, Tanner C, Castellano‐Smith A, et al. Validation of nonrigid image registration using finite‐element methods: Application to breast MR images. IEEE Trans Med Imaging 2003;22:238‐247. 12716000 10.1109/TMI.2002.808367

[88] Ashburner J. A fast diffeomorphic image registration algorithm. Neuroimage 2007;38:95‐113. 17761438 10.1016/j.neuroimage.2007.07.007

[89] Beg M, Miller M, Trouve A, Younes L. Computing large deformation metric mappings via geodesic flows of diffeomorphisms. Int J Comput Vis 2005;61:139‐157.

[90] Liu Z, Li Z, Qu J, et al. Radiomics of multiparametric MRI for pretreatment prediction of pathologic complete response to neoadjuvant chemotherapy in breast cancer: A multicenter study. Clin Cancer Res 2019;25:3538‐3547. 30842125 10.1158/1078-0432.CCR-18-3190

[91] Hirano M, Satake H, Ishigaki S, Ikeda M, Kawai H, Naganawa S. Diffusion‐weighted imaging of breast masses: Comparison of diagnostic performance using various apparent diffusion coefficient parameters. Am J Roentgenol 2008;191:689‐699. 22358015 10.2214/AJR.11.7093

[92] Dubois S, Peteri R, Menard M. Characterization and recognition of dynamic textures based on the 2d+t curvelet transform. Signal Image Video Process 2015;9:819‐831.

[93] Giannini V, Mazzetti S, Marmo A, Montemurro F, Regge D, Martincich L. A computer‐aided diagnosis (CAD) scheme for pretreatment prediction of pathological response to neoadjuvant therapy using dynamic contrast‐enhanced MRI texture features. Br J Radiol 2017;90:20170269. 28707546 10.1259/bjr.20170269 PMC5858803

[94] Lim I, Noh W, Park J, et al. The combination of fdg pet and dynamic contrast‐enhanced MRI improves the prediction of disease‐free survival in patients with advanced breast cancer after the first cycle of neoadjuvant chemotherapy. Eur J Nucl Med Mol Imaging 2014;41:1852‐1860. 24927797 10.1007/s00259-014-2797-4

[95] Pengel K, Koolen B, Loo C, et al. Combined use of ^1^8f‐fdg pet/ct and MRI for response monitoring of breast cancer during neoadjuvant chemotherapy. Eur J Nucl Med Mol Imaging 2014;41:1515‐1524. 24777490 10.1007/s00259-014-2770-2

[96] Wu G, Fan M, Zhang J, Zheng B, Li L. Prediction of response to neoadjuvant chemotherapy in breast cancer: A radiomic study. SPIE Med Imaging 2017;10138:101380E.

[97] Huang L, Fan M, Li L, Zhang J, Shao G, Zheng B. Association between dynamic features of breast DCE‐MR imaging and clinical response of neoadjuvant chemotherapy: A preliminary analysis. SPIE Med Imaging 2016;9789:97890Z.

[98] Tahmassebi A, Wengert GJ, Helbich TH, et al. Impact of machine learning with multiparametric magnetic resonance imaging of the breast for early prediction of response to neoadjuvant chemotherapy and survival outcomes in breast cancer patients. Invest Radiol 2019;54(2):110‐117. 30358693 10.1097/RLI.0000000000000518 PMC6310100

[99] Vandenberghe M, Scott M, Scorer P, Soderberg M, Balcerzak D, Barker C. Relevance of deep learning to facilitate the diagnosis of HER2 status in breast cancer. Sci Rep 2017;7:45938. 28378829 10.1038/srep45938 PMC5380996

[100] Huynh B, Antropova N, Giger ML. Comparison of breast DCE‐MRI contrast time points for predicting response to neoadjuvant chemotherapy using deep convolutional neural network features with transfer learning. SPIE Symp Med Imaging 2017;10134:101340U.

[101] Hoffman S, Lobbes M, Houben I, et al. Computer‐aided diagnosis of diagnostically challenging lesions in breast MRI: A comparison between a radiomics and a feature‐selective approach. SPIE Symp Comput Intellig 2016;98710H.

[102] Bickelhaupt S, Paech D, Kickingereder P, et al. Prediction of malignancy by a radiomic signature from contrast agent‐free diffusion MRI in suspicious breast lesions found on screening mammography. J Magn Reson Imaging 2017;46(2):604‐616. 28152264 10.1002/jmri.25606

[103] Prasann P, Tiwari P, Madabhushi A. Co‐occurence of local anisotropic gradient orientations (collage): A new radiomics descriptor. Sci Rep 2016;6:37241. 27872484 10.1038/srep37241 PMC5118705

[104] Anderson R, Li H, Li Y, Liu P, Giger M. Evaluating deep learning techniques for dynamic contrast‐enhanced MRI in the diagnosis of breast cancer. SPIE Med Imaging 2019;10950:UNSP1095006.

[105] Antropova N, Huynh B, Giger M. Performance comparison of deep learning and segmentation‐based radiomic methods in the task of distinguishing benign and malignant breast lesions on DCE‐MRI. SPIE Med Imaging 2017;10134:UNSP101341G.

[106] Antropova N, Huynh B, Giger M. A deep feature fusion methodology for breast cancer diagnosis demonstrated on three imaging modality datasets. Med Phys 2017;44:5162‐5171. 28681390 10.1002/mp.12453 PMC5646225

[107] Whitney H, Taylor N, Drukker K, et al. Additive benefit of radiomics over size alone in the distinction between benign lesions and luminal cancers on a large clinical breast MRI dataset. Acad Radiol 2019;26:202‐209. 29754995 10.1016/j.acra.2018.04.019 PMC9392156

[108] Zhou J, Zhang Y, Chang K, et al. Diagnosis of benign and malignant breast lesions on DCE‐MRI by using radiomics and deep learning with consideration of peritumor tissue. J Magn Reson Imaging 2019;51: 798–809. 31675151 10.1002/jmri.26981 PMC7709823

[109] Xiong Q, Zhou X, Liu Z, et al. Multiparametric MRI‐based radiomics analysis for prediction of breast cancers insensitive to neoadjuvant chemotherapy. Breast 2019;44:S68. 10.1007/s12094-019-02109-8 30977048

[110] Ji Y, Li H, Edwards A, et al. Independent validation of machine learning in diagnosing breast cancer on magnetic resonance imaging within a single institution. Cancer Imaging 2019;19:64. 31533838 10.1186/s40644-019-0252-2 PMC6751793

[111] Whitney HM, Li H, Ji Y, Liu P, Giger ML. Harmonization of radiomic features of breast lesions across international DCE‐MRI datasets. J Med Imaging 2020;7(1):012707. 10.1117/1.JMI.7.1.012707 PMC7056633 32206682

[112] Traverso A, Wee L, Dekker A, Gillies R. Repeatability and reproducibility of radiomic features: A systematic review. Int J Radiat Oncol Biol Phys 2018;102(4):1143‐1158. 30170872 10.1016/j.ijrobp.2018.05.053 PMC6690209

[113] Saha A, Yu XZ, Sahoo D, Mazurowski M. Effects of MRI scanner parameters on breast cancer radiomics. Exp Syst Appl 2017;87:384‐391. 10.1016/j.eswa.2017.06.029 PMC6176866 30319179

